# Successful long-term management of choroidal neovascularization secondary to angioid streaks in a patient with pseudoxanthoma elasticum: a case report

**DOI:** 10.1186/1752-1947-8-458

**Published:** 2014-12-22

**Authors:** Maria Cristina Savastano, Angelo Maria Minnella, Gaetano Zinzanella, Benedetto Falsini, Aldo Caporossi

**Affiliations:** Ophthalmology, Catholic University “Sacro Cuore – A. Gemelli”, Largo “A. Gemelli” 8, 00168 Rome, Italy

**Keywords:** Angioid streaks, Bevacizumab, Choroidal neovascularization, Pseudoxanthoma elasticum, Ranibizumab

## Abstract

**Introduction:**

We describe the long-term effectiveness and tolerability of intravitreal vascular endothelial growth factor inhibitor ranibizumab in a patient with pseudoxanthoma elasticum with bilateral macular choroidal neovascularization secondary to angioid streaks.

**Case presentation:**

A 54-year-old Caucasian man with history of heart disease presented with visual loss in his right eye. An examination revealed choroidal neovascularization and reduced visual acuity, while no abnormalities were seen in his left eye. He was diagnosed with angioid streaks associated with pseudoxanthoma elasticum. Off-label treatment with intravitreal bevacizumab once a month initiated in December 2007 was discontinued after 3 months due to lack of efficacy. In September 2008, the patient reported reduced visual acuity in his left eye and an examination revealed changes. Left eye treatment was initiated in October 2008 with a loading dose (three consecutive monthly intravitreal injections of ranibizumab 0.5mg/50μL) followed by 0.5mg/50μL followed by treatment as needed until May 2014. After 21 ranibizumab injections, an examination revealed angioid streaks and choroidal neovascularization in both eyes. His right eye showed retinal layer deterioration with outer limiting membrane and photoreceptor inner/outer segment junction involvement. His left eye had a smaller foveal scar, with other areas preserved. Visual acuity was stable in his treated left eye, but had deteriorated in his right eye. Ranibizumab treatment was well tolerated with no adverse events reported.

**Conclusions:**

In the present case, an as-needed regimen of ranibizumab after an initial loading dose, achieved maintenance of visual function and was well tolerated over a period of almost 6 years in a patient with pseudoxanthoma elasticum and high cardiovascular risk. As anti-vascular endothelial growth factor agents are associated with increased risk of systemic effects, particularly arterial thromboembolic events, following intravenous administration, the absence of serious thromboembolic or cardiovascular adverse events throughout the 6-year treatment period is particularly encouraging considering our patient’s high cardiovascular risk status.

## Introduction

Pseudoxanthoma elasticum (PXE) is a rare, inherited disease with typical ocular manifestations including angioid streaks (seen as breaks in the Bruch’s membrane) [1, 2]. A common and serious complication of angioid streaks is the development of macular choroidal neovascularization (CNV), which can result in significant and irreversible impairment of vision [3]. The introduction of intravitreally administered agents that inhibit vascular endothelial growth factor (VEGF) has improved outcomes in patients with CNV [3]. Ranibizumab – a recombinant humanized anti-VEGF-A monoclonal antibody antigen-binding fragment – is approved for intravitreal use for neovascular (wet) age-related macular degeneration and visual impairment due to diabetic macular edema, macular edema secondary to retinal vein occlusion or to CNV secondary to pathologic myopia [4]. Evidence suggests that anti-VEGF agents are effective in patients with angioid streak-related CNV associated with PXE [5–8].

We describe the long-term effectiveness and tolerability of the off-label use of anti-VEGF-A agent ranibizumab, in a patient with PXE with bilateral CNV secondary to angioid streaks.

## Case presentation

A 54-year-old Caucasian man with hypertension, diabetes mellitus, peripheral vascular insufficiency, and a history of ischemic heart disease, presented in December 2007 with significant visual loss in his right eye (RE) and the occurrence of metamorphopsia and central scotoma. His left eye (LE) was unaffected. He was in treatment with low-dose aspirin and oral antihyperglycemics and antihypertensives, and had undergone two triple coronary bypasses in the previous years (latest in 2004). His heart disease and diabetes were well compensated; his blood pressure and hemodynamic parameters were controlled.

Eye examinations (fundus photography, autofluorescence; fluorescein angiography; and optical coherence tomography, OCT) revealed the presence of CNV in his RE, while no abnormalities were seen in his LE. Best corrected visual acuity (BCVA) was 20 Early Treatment Diabetic Retinopathy Study (ETDRS) letters for his RE and 78 ETDRS letters for his LE. He was diagnosed with angioid streaks associated with PXE, which was confirmed by skin biopsy. Off-label treatment of his RE with intravitreal injection of bevacizumab (1.25mg/50μL) once a month was initiated in December 2007, but discontinued after 3 months in February 2008 due to lack of efficacy (no improvement in visual acuity).

**Figure 1:**
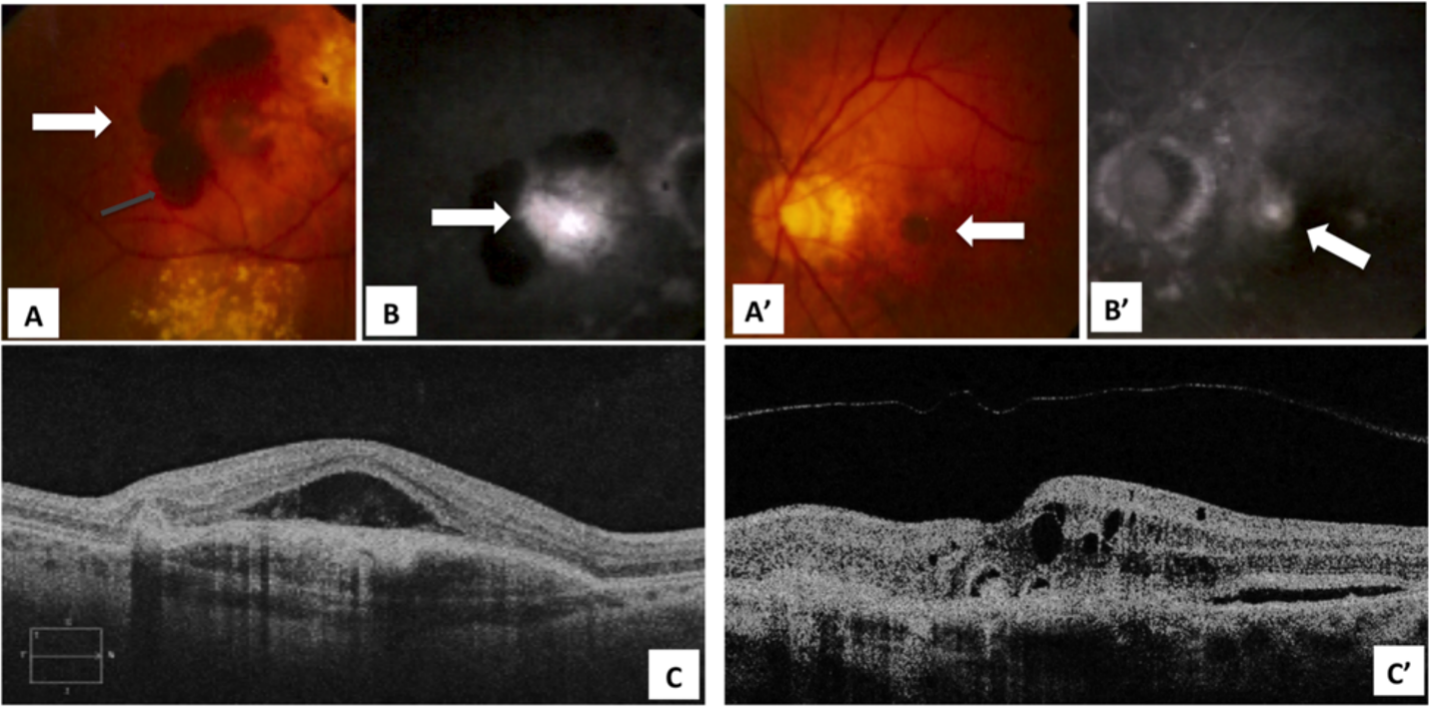
**Fundus photography and optical coherence tomography scans in the left eye (pre-ranibizumab) and the right eye (post-bevacizumab). (A, A’)** Fundus autofluorescence, **(B, B’)** fluorescein angiography, **(C, C’)** optical coherence tomography; **(A, B, C)** right eye; **(A’, B’, C’)** left eye. The left eye images are before ranibizumab treatment. The right eye images are after three intravitreal bevacizumab injections (September 2008). The color fundus images **(A-A’)** show the macular involvement by the choroidal neovascularization (white arrowheads) mainly in the right eye with severe bleeding (gray arrowhead). The choroidal neovascularization is visible in both eyes by fluorescein angiography (white arrows in **B** and **B’**). The optical coherence tomography shows retinal pigment epithelium detachment in the right eye with subretinal fluid over the neovascularization **(C)**. Initial retinal pigment epithelium interruption, intraretinal fluid and neuroretinal detachment are observable in the left eye **(C’)**.

In September 2008, He reported reduced visual acuity in his LE. Eye examinations (fundus photography, fluorescent angiography and OCT) revealed changes in his LE and confirmed angioid streaks and CNV in his RE (Figure 1). Photodynamic therapy was proposed but a second opinion was sought outside Italy. There, treatment of his LE with the anti-VEGF agent ranibizumab (unavailable in Italy at that time) was proposed and initiated in October 2008 with a loading dose (three consecutive monthly intravitreal injections of ranibizumab 0.5mg/50μL) followed by treatments as needed until May 2014 (endpoint of the present observation). His RE was not treated after February 2008.

Eye examinations – fundus photography, fluorescein angiography, B-scan OCT and en face OCT – were performed in May 2014. The reason for using en face OCT was to better investigate the “cystic-like appearance” of outer retinal tubulation (ORT) that may be confused with cystoid macular edema related to leakage from CNV. According to other authors [9], the presence of ORT during follow-up supports the concept that these structures are not a sign of ongoing neovascular activity and its presence, if not identified as ORT, can potentially lead to unnecessary treatment.

From October 2008 to May 2014, 21 intravitreal injections of ranibizumab were given in his LE: two in 2008, four in 2009 (one of which was part of the loading dose), three in 2010, four in 2011, four in 2012, three in 2013, and one in May 2014.

**Figure 2:**
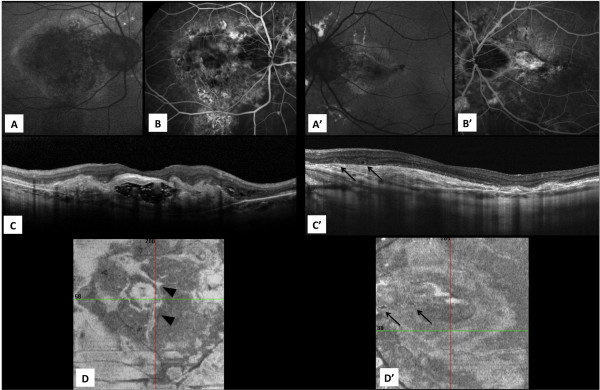
**Fundus photography (autofluorescence) and optical coherence tomography (OCT) images after almost 6 years of follow-up.** Left eye received intravitreal ranibizumab injections (loading dose during first three months, followed by treatment PRN) (May 2014). **(A, A’)** fundus autofluorescence, **(B, B’)** fluorescein angiography, **(C, C’)** B-scan OCT; **(D, D’)** En face OCT; **(A, B, C, D)** right eye; **(A’, B’, C’, D’)** left eye. Angioid streaks and choroidal neovascularization were visible in both eyes, while full posterior pole retinal involvement was observable only in the right eye **(A, B, C, D)**. In RE the extension of the scar was observable in en face scan **(D)** (black arrowhead). The LE B-scan OCT shows the neovascularization scar in the subfoveal region with external limiting membrane (ELM) and inner/outer photoreceptor junction preservation. The neuroretinal layers are observable over the lesion with no signs of inflammation. The B-scan and the en face OCT images **(C’, D’)** show the presence of outer retinal tabulation (ORT) along the posterior pole lesion (black arrows) that are generally observed in chronic disease with retinal rearrangement.

An eye examination in May 2014 revealed angioid streaks and CNV in both eyes (Figure 2). In his RE, a large fibrotic lesion was localized in the mainly posterior pole, while in his LE, the fovea was involved. His RE showed retinal layer deterioration with outer limiting membrane (OLM) and photoreceptor inner/outer segment junction involvement, while in his LE, the smaller foveal scar preserved the photoreceptor junction, the OLM and the retinal layers (Figure 2).

His visual acuity was stable in his ranibizumab-treated LE but had further decreased in his RE. Intraocular pressure was normal (16mmHg) in both eyes. Ranibizumab treatment was well tolerated with no adverse events reported throughout the 6-year treatment period.

## Discussion

In our patient, early intravitreal ranibizumab administration to his less severely affected LE preserved moderate visual function over the entire observation period, whereas in his first affected RE, bevacizumab had failed to preserve visual function. This may be explained by the fact that the CNV in his RE was in an advanced stage, already associated with irreversible vision impairment. Published evidence suggests that to achieve optimal results in the management of CNV in PXE, anti-VEGF therapy should be started as early as possible [6]. In a case report of 5-year ranibizumab in PXE, a 51-year-old patient achieved complete disease stabilization with 12 intravitreal injections in the first year [10]. During the subsequent 4 years, he received two additional injections for mild recurrence only and his LE vision remained stable over the entire observation period.

Our patient was treated with a different regimen of ranibizumab loading dose for 3 months and then as needed, with a mean interval between injections of 3.5 months, so our findings demonstrate the effectiveness of a regimen with longer between-dose intervals. These observations support those from two case series of PXE treated with ranibizumab: >85% of patients had improved or stable BCVA [7, 8]. Notably, in our patient, during the last 3 years, injection frequency has decreased progressively to only one during the first 5 months of 2014 given as a precautionary measure.

The absence of serious thromboembolic or cardiovascular (CV) events throughout the 6-year treatment period is noteworthy considering the high CV risk of our patient. Systemic effects, particularly arterial thromboembolic events, following intravenous anti-VEGF agents, have raised concern regarding the use of these agents in patients with PXE due to the high CV risk. Ranibizumab with its shorter systemic half-life versus other anti-VEGF agents may have safety advantages in this population [11–14].

Finally, our case also highlights the benefits of en face OCT [15], which allowed for transversal assessment of photoreceptor involvement and outer retinal tubulation of the outer nuclear retinal layers (Figures 2D and 2D’).

## Conclusions

In conclusion, in a patient with PXE and high CV risk, a 6-year ‘as-needed’ regimen of ranibizumab (after an initial loading dose) achieved maintenance of visual function and was well tolerated. As anti-VEGF agents are associated with increased risk of systemic effects, particularly arterial thromboembolic events, following intravenous administration, the absence of serious thromboembolic or CV adverse events throughout the 6-year treatment period is particularly encouraging considering the high CV risk status of this patient.

## Consent

Written informed consent was obtained from the patient for publication of this case report and accompanying images. A copy of the written consent is available for review by the Editor-in-Chief of this journal.

## Abbreviations

BCVA: Best corrected visual acuity
CNV: Choroidal neovascularization
CV: Cardiovascular
ETDRS: Early Treatment Diabetic Retinopathy Study
LE: Left eye
OCT: Optical coherence tomography
OLM: Outer limiting membrane
ORT: Outer retinal tubulation
PXE: Pseudoxanthoma elasticum
RE: Right eye
VEGF: Vascular endothelial growth factor.
